# Exploring Metabolic Pathways and Gene Mining During Cotton Flower Bud Differentiation Stages Based on Transcriptomics and Metabolomics

**DOI:** 10.3390/ijms26052277

**Published:** 2025-03-04

**Authors:** Miaoqian Yang, Wenjie Li, Xiaokang Fu, Jianhua Lu, Liang Ma, Hantao Wang, Hengling Wei

**Affiliations:** National Key Laboratory of Cotton Bio-Breeding and Integrated Utilization, Institute of Cotton Research, Chinese Academy of Agricultural Sciences, Anyang 455000, China; y964565060@163.com (M.Y.); 18699550611@163.com (W.L.); xkang_2010@163.com (X.F.); lujh212760@163.com (J.L.); maliang3417@163.com (L.M.)

**Keywords:** early-maturing cotton, flower bud differentiation, metabolome, transcriptome, *GhTYDC*

## Abstract

Cotton is regarded as one of the significant economic crops in China, and its earliness is defined as one of the crucial traits influencing fiber quality and yield. To study the physiological and biochemical mechanisms related to early-maturing traits of cotton, cotton shoot apexes at the one-leaf, three-leaf, and five-leaf stages of the early-maturing cotton CCRI50 and late-maturing cotton Guoxinmian11 were collected for transcriptome sequencing and metabolomics, respectively. A total of 616, 782, and 842 differentially expressed genes (DEGs) at the one-leaf stage, three-leaf stage, and five-leaf stage were obtained through transcriptome sequencing, respectively. The metabolic detection results showed that 68, 56, and 62 differential metabolites (DMs) were obtained in the three periods, respectively. A total of 10 DMs were detected simultaneously from the one-leaf to five-leaf stage, 4 of which were phenolic acids and down-regulated in the early maturing variety CCRI50. A combined transcriptomic and metabolomic analysis revealed that phenylpropanoid biosynthesis, tyrosine metabolism, and phenylalanine metabolism might be important metabolic pathways in cotton bud differentiation. *GhTYDC-A01* was identified in both the tyrosine metabolism and phenylalanine metabolism pathways, and it was highly expressed in pistils. To investigate the function of this gene in flowering, we overexpressed it in *Arabidopsis thaliana*. Compared to the wild type, the flowering time of the overexpression of *GhTYDC-A01* in *Arabidopsis* was delayed. This study provides valuable resources and new insights into the relationship between metabolites and early-maturing cotton.

## 1. Introduction

Cotton, as the main raw material for the textile industry, is an important crop and strategic material related to the economy of the world [1]. Flowering is a crucial developmental transition stage in the life cycle of cotton, and it is also one of the criteria for determining its early maturity [2]. Early-maturing cotton has a shorter growth cycle and stronger adaptability compared to traditional cotton, which can result in higher profits and increase multiple crop indexes [3]. Flower bud differentiation plays an important role in early-maturing cotton. The flower bud differentiation of plants is influenced and regulated by various internal and external factors [4]. Internal factors include genotype, endogenous hormones, and carbohydrates [5,6], and external factors mainly include light, water, plant growth regulators, and nutrients [7,8]. For the study of plant flower bud differentiation, it is necessary to understand and explore this life activity process from multiple perspectives and aspects.

Transcriptomics can not only reflect gene expression as a whole but also deeply explore the laws of biological life activities at the molecular level [9]. Several differentially expressed genes (DEGs) related to the flowering of *Lycoris radiata* were obtained using transcriptome sequencing, and they can interact with SQUAMOSA-PROMOTER BINDING PROTEIN-LIKE (SPL), auxin, gibberellin, and several transcription factors to promote the flowering of *L. radiata* [10]. DEGs related to flowering were identified in rapeseed, and it was found that the expression of these DEGs changes with the variation of flowering traits [11]. In addition, some floral meristem genes, such as *CpAP1*, *CpAP3*, *CpPI*, *CpAGL6*, *CpSEP1*, *CpLFY*, and *CpUFO*, were also found to be involved in the development of cherry petals and stamens through the analysis of the transcriptome [12]. Using transcriptomics, 28,508 DEGs associated with the development of *Erythronium japonicum* at various floral stages were identified, along with 44 DEGs that were consistently regulated across all stages [13]. In addition, there are studies on floral bud differentiation in species such as *Camellia sinensis* [14], *Jatropha curcas* [15], *Castanea mollisima* [16], and *Juglans regia* [17].

With the development of systems biology, metabolomics has been widely applied in multiple fields [18,19], enabling the visualization of metabolite changes within plants [20,21]. The integration of transcriptomics and metabolomics provides a novel approach for further exploring the relationship between genes and metabolites [22,23,24]. For example, in chili peppers [9], tomatoes [25], blackberries [26], and beans [27], key genes have been identified within regulatory pathways [28,29]. Gibberellins (GAs) and brassinolide (BR) are key plant hormones involved in regulating plant growth and development [30,31] and can promote plant flowering [32]. It was found that the expression of DEGs changes with the change in GA content, thus affecting the flowering of Chinese cabbage [33]. Mao et al., 2022, found that six major synthase-encoding genes were up-regulated during the critical period of flower bud differentiation, leading to an increase in BR content and promoting the flowering of *Pak Choi* [34]. The content of metabolites in the sucrose and trehalose metabolic pathways significantly increased during the second flower bud differentiation period of *Magnolia*, and the expression of related genes also significantly increased [35]. The active regulatory network, in conjunction with photosynthesis and phenylpropanoid biosynthesis, played a crucial role in inducing the expression of flowering-related genes, thereby promoting the flowering process of *Ferula xinjiangensis* [36]. The transcriptome and metabolome were correlated in tea samples of different months, and 13 genes associated with terpenoid metabolism were discovered [37].

The rational use of omics technology can increase our understanding of plant growth and development mechanisms [38,39]. At present, there is relatively little research on the metabolism of the cotton flower bud differentiation stage. This study is based on a metabolomics analysis of the metabolic differences between early-maturing and late-maturing cotton varieties, combined with transcriptomics, to explore the metabolic substances regulating cotton flower bud differentiation, identify their regulatory genes, and verify the functions of these genes.

## 2. Results

### 2.1. Identification and Enrichment Analysis of the DEGs Between Two Varieties

**Figure 1:**
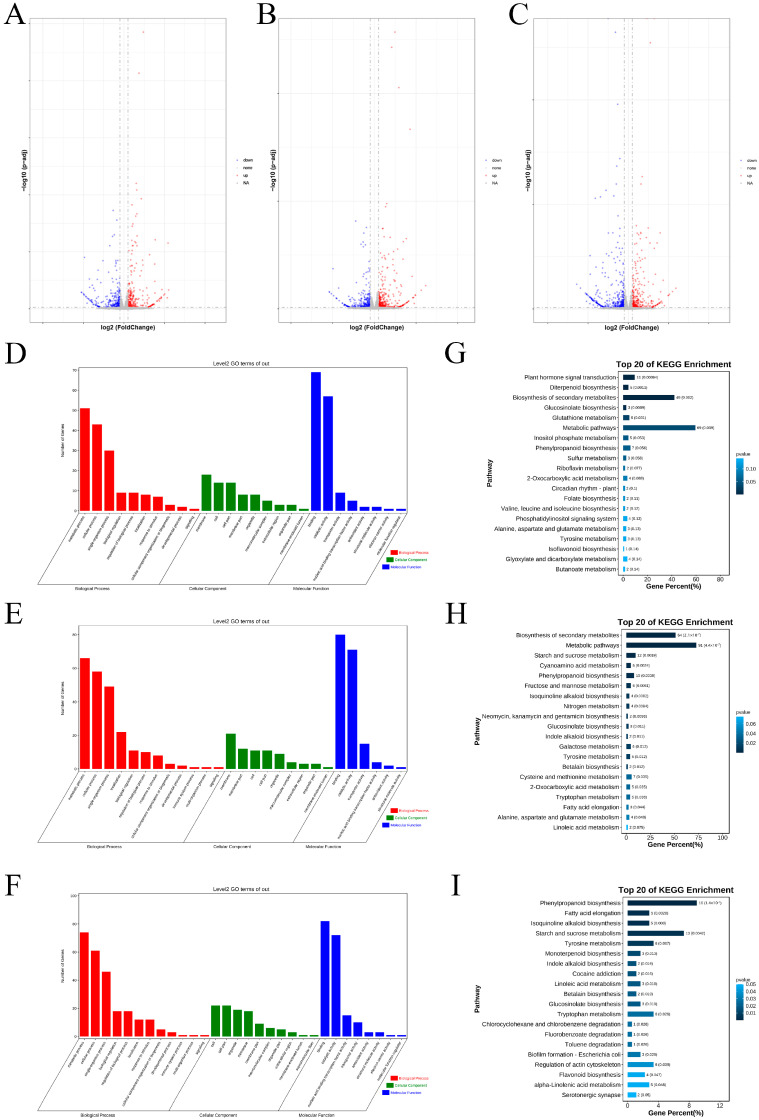
Identification and enrichment analysis of the DEGs between two varieties. (**A**–**C**) Volcanic diagram of DEGs at the 1-leaf, 3-leaf, and 5-leaf stages, successively (FDR ≤ 0.05, |log2FoldChange| ≥ 1). (**D**–**F**) GO enrichment analysis of all DEGs at the 1-leaf, 3-leaf, and 5-leaf stages, successively. (**G**–**I**) KEGG enrichment analysis of DEGs at the 1-leaf, 3-leaf, and 5-leaf stages, successively. The darker the color, the more significant the enrichment of DEGs in the pathway; *p*-value > 0.05.

A total of 616 DEGs were obtained at the one-leaf stage, with 308 genes up-regulated and 308 genes down-regulated (Figure 1A, Table S1). There were 782 DEGs at the three-leaf stage, of which 448 genes were up-regulated and 334 genes were down-regulated (Figure 1B, Table S1). At the five-leaf stage, a total of 843 DEGs were identified, including 364 up-regulated genes and 479 down-regulated genes (Figure 1C, Table S1). In conclusion, the number of DEGs increased with the progress of flower bud differentiation, in which the number of up-regulated genes was the highest at the three-leaf stage, while the number of down-regulated genes was the highest at the five-leaf stage.

The Gene Ontology (GO) enrichment analysis of DEGs was divided into three main categories: Biological Process (BP), Molecular Function (MF), and Cellular Component (CC). At the one-leaf stage, the BP category was enriched with 10 sub-categories, with the highest number of genes found in the metabolic process (Figure 1D). In the BP of the three-leaf and five-leaf stages, 232 and 252 genes were enriched, respectively, with a high proportion of them being in the metabolic process, cellular process, and single-organism process (Figure 1E,F). At the three periods, the two pathways with the highest gene enrichment in MF were binding and catalytic activity. The two pathways with the most enriched genes in CC were membrane and cell.

The Kyoto Encyclopedia of Genes and Genomes (KEGG) analysis revealed that, at the one-leaf and three-leaf stages, the most enriched regulatory pathway was the metabolic pathway, followed by the biosynthesis of secondary metabolites (Figure 1G,H). At the five-leaf stage, the pathway with the largest proportion was phenylpropanoid biosynthesis, followed by starch and sucrose metabolism, which accounted for the second-largest proportion (Figure 1I). Among these, the metabolic pathway that enriched in the five-leaf stage contained the largest number of genes across all stages and pathways.

### 2.2. Verification of DEGs Based on qRT-PCR

**Figure 2:**
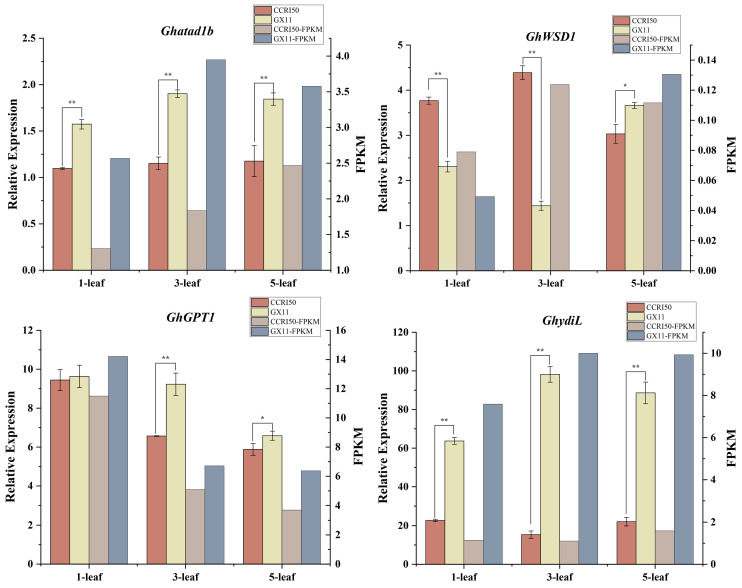
qRT-PCR and RNA-seq results of 4 selected DEGs between two varieties (* *p* < 0.05, ** *p* < 0.01).

We used qRT-PCR to detect the relative expression levels of four randomly selected DEGs (*Ghatad1b*, *GhWSD1*, *GhGPT1*, and *Ghydil*) between CCRI50 and Guoxinmian11. The results indicated that the expression levels of these four genes varied significantly between the two cotton varieties. Combined with the RNA-seq results, the relative expression levels of the genes in the flower buds as determined by qRT-PCR were largely consistent with the expression trends observed in the RNA-seq data (Figure 2).

### 2.3. Identification of Differential Metabolites (DMs) Between Two Cotton Varieties

**Figure 3:**
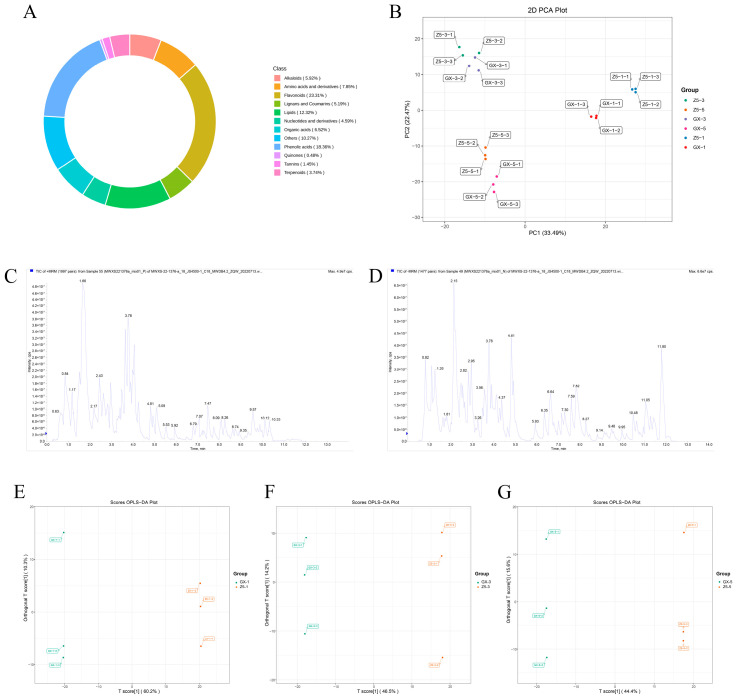
Metabolomic characteristics of DMs. (**A**) Circular diagram of metabolite category composition. Each color represents a DM category, and the area of each segment corresponds to the proportion of that category. (**B**) PCA plot. PC1 represents the first principal component. PC2 represents the second principal component. The percentages indicate the proportion of variance explained by each principal component. Each point on the plot represents a sample, and samples from the same group are represented using the same color. Group refers to the grouping of samples. (**C**) Positive Ion Mode TIC for QC samples. (**D**) Negative Ion Mode TIC for QC samples. (**E**–**G**) The OPLS-DA score plot for the 1-leaf, 3-leaf, and 5-leaf stages, successively. The horizontal axis represents the predicted principal components, and the difference between groups can be seen in the direction of the horizontal axis. The vertical axis represents the orthogonal principal components, and the difference within the group can be seen in the direction of the vertical axis.

A total of 828 metabolites were identified in the metabolic analysis (Figure 3A), including 193 flavonoids, 152 phenolic acids, 102 lipids, 65 amino acids and derivatives, 54 organic acids, 54 saccharides, 49 alkaloids, 43 lignans and coumarins, 38 nucleotides and derivatives, 31 terpenoids, 13 vitamins, 12 tannins, 5 alcohol compounds, 4 quinones, 4 lactones, and 9 others.

The Principal Component Analysis (PCA) (Figure 3B), Total Ion Current (TIC) (Figure 3C,D), and Orthogonal Partial Least Squares Discriminant Analysis (OPLS-DA) score plots (Figure 3E–G) of the quality control (QC) samples all showed that the experiment was highly reliable, and the metabolic substances differed significantly between early-maturing and late-maturing cotton varieties at different stages.

**Figure 4:**
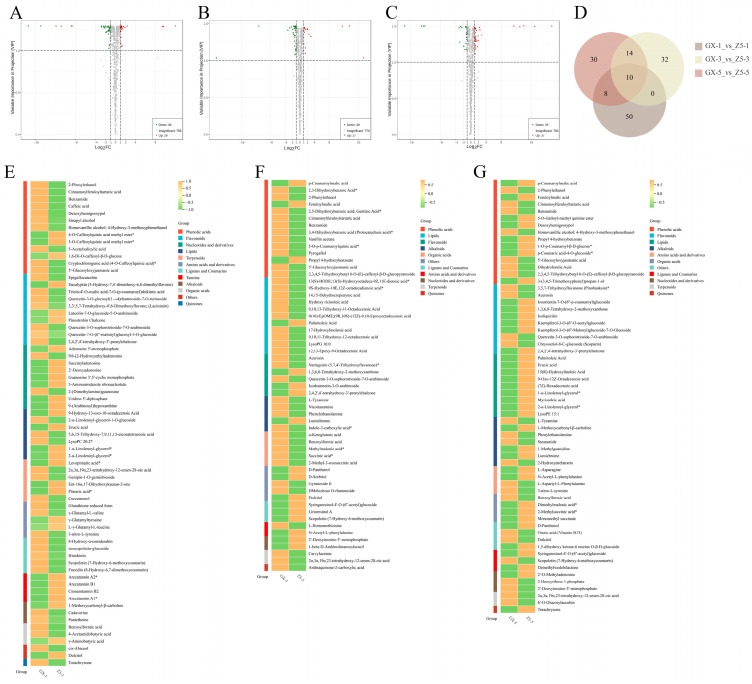
Volcano plots of DMs for three periods and clustering analysis results. (**A**–**C**) Volcanic diagram of DMs at the 1-leaf, 3-leaf, and 5-leaf stages, successively. (**D**) Venn diagram of DMs at the three periods. (**E**–**G**) Cluster analysis results of DMs at the 1-leaf, 3-leaf, and 5-leaf stages, successively. Orange indicates high abundance, and green indicates low abundance (* isomer).

The results of the differential metabolite (DM) screening showed that there were 29 up-regulated DMs and 39 down-regulated DMs at the one-leaf stage (Figure 4A). At the three-leaf stage, there were 17 up-regulated DMs and 39 down-regulated DMs (Figure 4B). At the five-leaf stage, there were 37 up-regulated DMs and 25 down-regulated DMs (Figure 4C). Among them, the number of up-regulated DMs at the three-leaf stage was the smallest at the three stages.

### 2.4. Cluster Analysis of DMs

A total of 68 DMs were clustered at the one-leaf stage. Except for 5-O-caffeoylquinic acid methyl ester, 4-O-caffeoylquinic acid methyl ester, and 1,6-Di-O-caffeoyl-β-D-glucose, the contents of other components in phenolic acids were down-regulated. The contents of three substances in flavonoids were higher in CCRI50. Most of nucleotides and derivatives were up-regulated except for N6-(2-hydroxyethyl) adenosine and 2-(Dimethylamino) guanosine. There were four metabolites up-regulated in lipids. There were three terpenoids up-regulated. All lignans and coumarins were down-regulated. All tannins were up-regulated (Figure 4E). At the three-leaf stage, 56 DMs were clustered. Among the phenolic acids, the contents of p-coumaroylmalic acid, feruloylmalic acid, and propyl 4-hydroxybenzoate were up-regulated, while other components were down-regulated. In the lipids, only palmitoleic acid was up-regulated. All five organic acids were down-regulated. In CCRI50, the content of all lignans, coumarins, and nucleotides and derivatives were up-regulated compared to Guoxinmian11. All terpenoids and quinones were down-regulated (Figure 4F). There were 62 DMs at the five-leaf stage, and 7 metabolites out of 15 phenolic acids were up-regulated. Six metabolites were up-regulated among the ten flavonoids. Lipids and organic acids showed an overall up-regulation. All terpenoids were down-regulated (Figure 4G).

Figure 4D shows that a total of 10 DMs were detected from the one-leaf to five-leaf stages: 4 phenolic acids, 2 flavonoids, 1 organic acid, 1 terpenoid, 1 lignan and coumarin, and 1 carbohydrate. And all four phenolic acids were down-regulated at the three stages in CCRI50. The results suggested that phenolic acids might be involved in the process of cotton bud differentiation and thus affect cotton flowering.

### 2.5. KEGG Analysis of DMs

**Figure 5:**
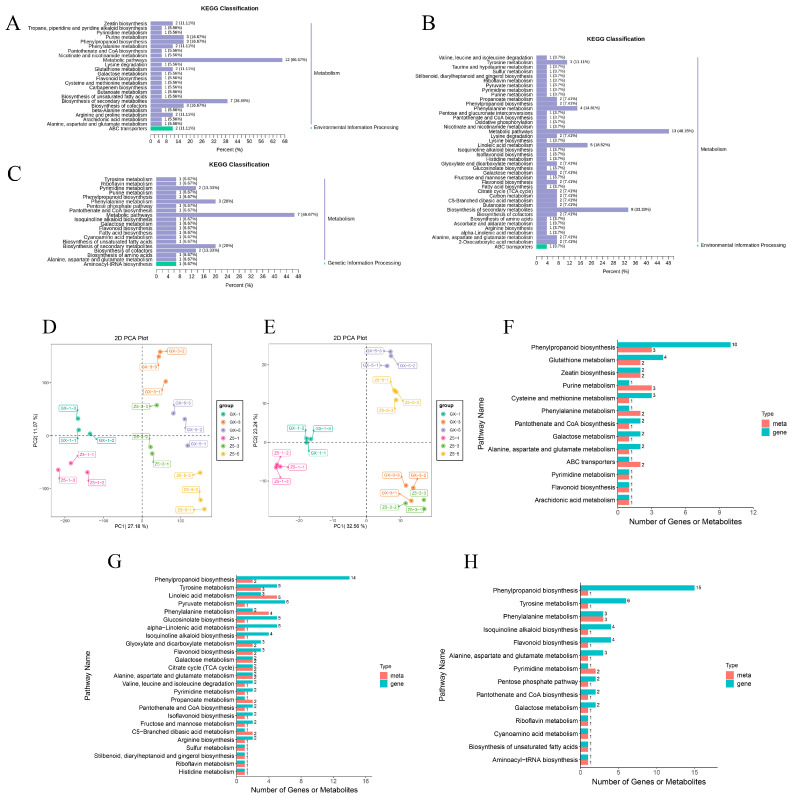
KEGG enrichment analysis of DMs in three periods and correlation analysis results. (**A**–**C**) KEGG enrichment analysis of DMs at the 1-leaf, 3-leaf, and 5-leaf stages. (**D**) PCA chart of transcriptome. (**E**) PCA chart of metabolome. (**F**–**H**) Bar chart of KEGG enrichment analysis for combined multi-omics data at the 1-leaf, 3-leaf, and 5-leaf stages, successively. The red and green bars represent the metabolome and transcriptome, respectively.

At the one-leaf stage, it was found that 20 out of 68 DMs were enriched via the KEGG in 24 pathways (Figure 5A), of which metabolic pathways, biosynthesis of secondary metabolites, phenylpropanoid biosynthesis, purine metabolism, and biosynthesis of cofactors were the main enrichment pathways. And most of the metabolites were nucleotides and derivatives, organic acids, and phenolic acids, representing a large proportion in the five pathways (Table S2). At the three-leaf stage, 27 DMs were enriched in 42 pathways (Figure 5B), and metabolic pathways, biosynthesis of secondary metabolites, linoleic acid metabolism, and phenylalanine metabolism were the main enrichment pathways. Organic acids, lipids, phenolic acids, and flavonoids accounted for a significant proportion in those four pathways (Table S3). At the five-leaf stage, 15 out of 62 DMs were enriched in 20 pathways. And metabolic pathways, phenylalanine metabolism, and biosynthesis of secondary metabolites were the main pathways (Figure 5C and Table S4). The results showed that metabolic pathways, biosynthesis of secondary metabolites, and phenylalanine metabolism play important roles from the one-leaf to five-leaf stages (Figure 5A–C).

### 2.6. Correlation Analysis Based on Transcriptome and Metabolome

In order to better understand the physiological and molecular mechanisms of cotton bud differentiation, a correlation analysis was performed based on the transcriptome and metabolome. All Pearson correlation calculation (Pcc) results of differential metabolites and differential genes with correlation coefficients greater than 0.8 were selected to draw the cluster heatmap of correlation coefficients (Figure S1, Table S5). The results showed that the correlation between DEGs and DMs was at a high level at the three stages. The PCA results showed that there were differences among the groups in both the transcriptome (Figure 5D) and metabolome (Figure 5E), which indicated that the experiment was highly reliable.

At the one-leaf stage, DEGs and DMs were enriched in 13 pathways (Figure 5F). Phenylpropanoid biosynthesis, glutathione metabolism, purine metabolism, and cysteine and methionine metabolism were the main pathways. The phenylpropanoid biosynthesis pathway included 10 DEGs and three DMs. The three DMs were phenolic acids and were down-regulated. The 10 DEGs were all related to the metabolic pathway. At the three-leaf stage, DEGs and DMs were enriched in 31 pathways (Figure 5G). Phenylpropanoid biosynthesis, tyrosine metabolism, linoleic acid metabolism, pyruvate metabolism, phenylalanine metabolism, glucosinolate biosynthesis, and alpha-Linolenic acid metabolism were the main pathways. At the five-leaf stage, DEGs and DMs were enriched in 14 pathways (Figure 5H). Phenylpropanoid biosynthesis, tyrosine metabolism, phenylalanine metabolism, isoquinoline alkaloid biosynthesis, and flavonoid biosynthesis were the main pathways. The phenylpropanoid biosynthesis pathway contained 15 DEGs, 11 of which were down-regulated, indicating continued negative regulation of phenylpropanoid synthesis. The pathway with the most DMs was phenylalanine metabolism, which contained three DMs of distinct types, including phenolic acid, organic acid, and amino acid. The results of the enrichment analysis showed that phenylpropanoid biosynthesis, tyrosine metabolism, and phenylalanine metabolism might be important metabolic pathways in cotton bud differentiation.

### 2.7. Identification of Candidate Genes Based on Transcriptome and Metabolome

**Figure 6:**
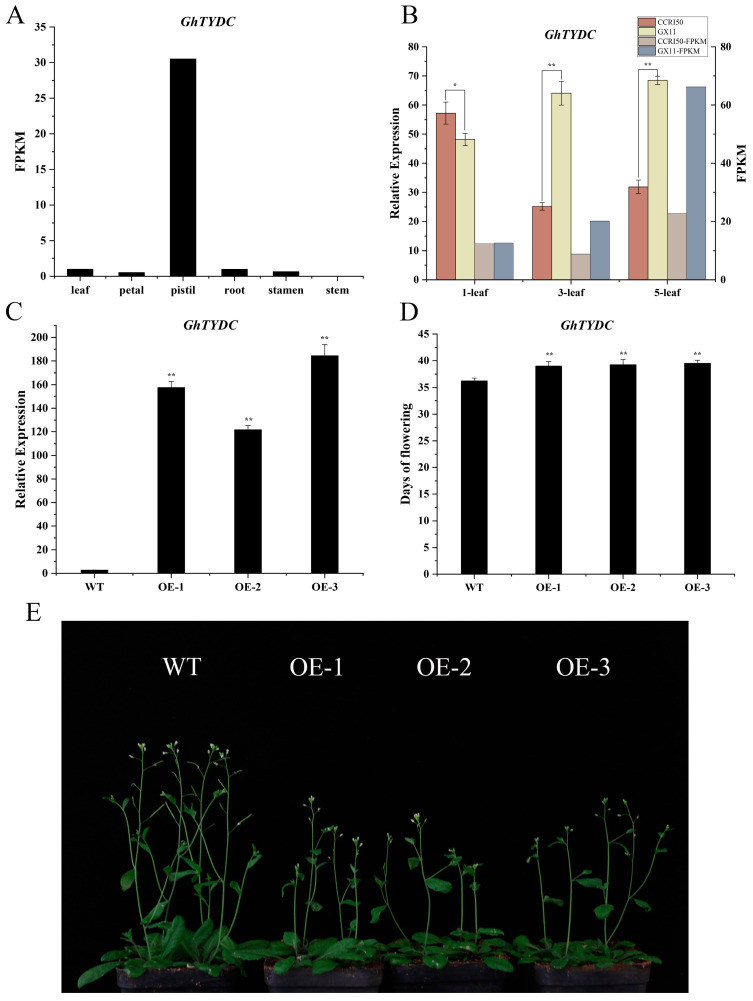
Analysis of *GhTYDC-A01* expression pattern and phenotype observation. (**A**) Expression patterns of *GhTYDC-A01* in various tissues of TM-1. (**B**) Expression patterns of *GhTYDC-A01* in CCRI50 and Guoxinmian11. (**C**) The expression levels of *GhTYDC-A01* in transgenic plants were significantly higher than those in WT plants (* *p* < 0.05, ** *p* < 0.01). (**D**) The flowering time of *GhTYDC-A01* in transgenic plants was significantly later than that in WT plants (* *p* < 0.05, ** *p* < 0.01). (**E**) Overexpression of *GhTYDC-A01* delayed the flowering time in *Arabidopsis*.

In the previous studies, flower bud differentiation occurred at the three-leaf stage in early-maturing cotton varieties but at the five-leaf stage in late-maturing cotton varieties [40]. The result of the correlation analysis of the transcriptome and metabolome showed that phenylpropanoid biosynthesis, tyrosine metabolism, and phenylalanine metabolism were the main pathways from the one-leaf to five-leaf stages. And the gene *GH_A01G0164* was identified in both the tyrosine metabolism and phenylalanine metabolism pathways. It was found to be the homologous gene of *AtTYDC* and was named *GhTYDC-A01*. Based on the tissue transcriptome data of TM-1 [41], *GhTYDC-A01* was highly expressed in pistils (Figure 6A).

### 2.8. Overexpression of GhTYDC-A01 Leads to Delayed Flowering Time in Arabidopsis

Comparing the expression levels of *GhTYDC-A01* at the three stages between CCRI50 and Guoxinmian11, it was found that *GhTYDC-A01* was higher in CCRI50 at the one-leaf stage and higher in Guoxinmian11 at the three-leaf and five-leaf stages (Figure 6B). The qRT-PCR results showed that the expression levels of *GhTYDC-A01* in transgenic plants were significantly higher than those in WT plants (Figure 6C). Compared to the wild-type *Arabidopsis*, overexpression of *GhTYDC-A01* delayed the flowering time (Figure 6D,E). This suggested that *GhTYDC-A01* may delay the flower bud differentiation and inhibit the flowering time in cotton.

## 3. Discussion

Cotton is one of the most important economic crops in China and around the world. The application of omics technologies provides new insights into understanding the molecular mechanisms underlying cotton flower bud differentiation. In this experiment, shoot apexes from two cotton cultivars, CCRI50 and Guoxinmian11, at the one-leaf, three-leaf, and five-leaf stages were collected for transcriptomic sequencing. The GO analysis of the three stages revealed that most DEGs were enriched in the metabolic process, single-organism process, and cellular process pathways. In addition, we found that after the initiation of flower bud differentiation in CCRI50 at the three-leaf stage, most of the genes are exercising the functions of binding and catalytic activity, and after the advancement of metabolic processes, cellular processes, and the process of a single organism, cells and some membrane substances are gradually formed, which promote the process of flower bud differentiation and make the shoot apexes begin to grow.

In recent years, studies have shown that secondary metabolites play various roles in plants, including regulating growth and development [42,43], interacting with the environment [44], and enhancing plant adaptation to both abiotic and biotic stresses [45,46]. Therefore, it is hypothesized that secondary metabolites may also play an important role in the growth and development of cotton. Among these, the metabolic pathways enriched at the five-leaf stage contained the largest number of genes across all stages and pathways, which may be attributed to the increased metabolic activity and expression of related flowering genes following the initiation of flower bud differentiation in cotton. We also found that most of the DEGs were enriched in regulatory pathways potentially related to cotton growth and development, such as biosynthesis of secondary metabolites, phenylpropanoid biosynthesis, and other metabolic pathways. In the study of peach [47] and *Chrysanthemum morifolium* [48] flower bud differentiation, it was found that the levels of phenolic acids and flavonoids gradually decreased. At different stages, DEGs were mainly concentrated in the biosynthesis pathway of phenylpropanoid and negatively regulated this synthesis. Research has shown that the biosynthesis of phenylpropanoid is crucial for inducing the expression of flowering-related genes in leaves of *F. sinkianensis* [36].

The KEGG analysis of DMs showed that metabolic pathways, biosynthesis of secondary metabolites, and phenylalanine metabolism play important roles from the one-leaf to five-leaf stages. The main metabolites in these three pathways included organic acids, phenolic acids, and flavonoids. The analysis of the 10 DMs that coexist in the three periods revealed that the main metabolites were still phenolic acids, flavonoids, and organic acids and carbohydrates. And all phenolic acids showed down-regulation at the three stages in CCRI50. Carbohydrates can act as induction signals for plant flowering [49], promote the elongation of pollen tubes [50,51], and facilitate petal opening by regulating osmotic balance [52]. Flavonoids are essential factors in the formation of pollen tubes [53,54,55]. Phenolic acids can affect plant flowering by regulating auxin [56]. Organic acids serve as crucial intermediates in metabolic pathways, facilitating the storage and provision of carbon sources and thereby ensuring adequate energy supply for the flowering process [57]. Therefore, we speculated that phenolic acids, organic acids, and flavonoids affected metabolism and synthesis processes during flower bud differentiation, thereby regulating cell growth and stem and leaf elongation.

The results of the transcriptional and metabolic correlation analysis showed that phenylpropanoid biosynthesis, tyrosine metabolism, and phenylalanine metabolism might be important metabolic pathways in cotton bud differentiation. These three metabolic pathways are also involved in the flowering process of many crops, such as rose [58], stevia [59], safflower [60], and faba bean [61]. And the gene *GH_A01G0164* was identified in both tyrosine metabolism and phenylalanine metabolism pathways [41], which was found to be the homologous gene of *AtTYDC* and was named *GhTYDC-A01*. Research has shown that the application of tyrosine could alter the sterility of treated tobacco and restore its flowering [62]. *GhTYDC-A01* is highly expressed in pistils, so we speculated that *GhTYDC* might be involved in the flower bud differentiation of cotton. The activity of TYDC can affect the levels of tyramine and phenylalanine simultaneously [63] and participates in the transformation and synthesis of amino acids in plants [64]. TYDC can also alter secondary metabolic pathways in plants [65,66]. During plant development, endogenous hormones play a crucial role, as they not only regulate cellular responses at the cellular level but also control the expression of certain genes at the molecular level [67]. Aux/IAA is a key regulatory factor in the auxin signaling pathway [68], and TYDC can participate in the synthesis of IAA and other plant metabolic processes [69]. *RvTYDC* can catalyze the production of IAA from tryptophan, thereby affecting plant development [70,71]. In the early-maturing and late-maturing materials, the relative expression level of *GhTYDC-A01* was higher at the three-leaf and five-leaf stages in Guoxinmian11. Overexpression of *GhTYDC-A01* resulted in a slower growth rate of *Arabidopsis* compared to the wild type. Numerous studies have demonstrated that the overexpression of a specific gene in both *Arabidopsis* and cotton leads to analogous phenotypic outcomes, for example, *GhPIF4a* [72], *GhFT* [73], and *GhAP1* [74]. Based on this, we speculated that overexpression of *GhTYDC-A01* has a certain inhibitory effect on cotton flower bud differentiation and flowering time. The flowering time is closely related to the early maturity of cotton [40]. In future studies, transgenic plants of *GhTYDC-A01* can be obtained in cotton using overexpression and CRISPR-Cas9 gene editing technology, providing an important source of genes and materials for cotton molecular breeding.

## 4. Materials and Methods

### 4.1. Experimental Materials

The cotton varieties used in this study were CCRI50 and Guoxinmian11. CCRI50, designated as the experimental group, has a plant height of 70–75 cm and a growing period of 104 days, making it an early-maturing cotton variety. Guoxinmian11, used as the control group, is considered a late-maturing variety with a growing period of 123 days.

The experimental samples in this study were cotton shoot apexes collected at three key growth stages: the time when the first leaf was fully expanded, the time when the third leaf was fully expanded, and the time when the fifth leaf was fully expanded. Sampling was performed at these 3 stages. For each sample, 10 plants with consistent growth were selected and pooled together as the first biological replicate, with three biological replicates in total. After sampling, the material was immediately frozen in liquid nitrogen for at least 2 min and then stored at −80 °C.

### 4.2. Analysis of Transcriptome Data

The transcriptome data of shoot apexes at the 1-leaf, 3-leaf, and 5-leaf stages of CCRI50 and Guoxinmian11 have been published and were downloaded from our previous study [40]. The TM-1 reference genome (*Gossypium hirsutum*, ZJU) [75] was downloaded from CottonFGD (https://cottonfgd.org/about/download.html, accessed on 10 October 2022). The raw reads generated were processed using Perl scripts and the FASTX Toolkit (https://github.com/agordon/fastx_toolkit/releases, accessed on 10 October 2022), which included the removal of adapter sequences and reads with more than 10% Ns, while retaining reads with a quality score of Q30. After removing low-quality reads, Illumina sequencing reads were mapped to the TM-1 reference genome using TopHat [76] with default settings for parameters. FPKM was used to quantitatively estimate the value of gene expression. DESeq2 [77] was used to analyze the DEGs, and FDR ≤ 0.05, |log2FoldChange| ≥ 1 were considered to be DEGs between CCRI50 and Guoxinmian11. CCRI50 was compared with the same stages of Guoxinmian11.

AgriGO [78] was used for the GO analysis. The correct error detection rate of GO terminology is 0.05, which is considered to be significantly enriched.

R was used for the KEGG analysis, and clusterProfiler was used for the KEGG pathway enrichment analysis of DEGs.

### 4.3. Total RNA Extraction and First-Strand cDNA Synthesis

Total RNA was extracted using the FastPure^®^ Universal Plant Total RNA Isolation Kit (Vazyme, Nanjing, China). First-strand cDNA was synthesized using the PrimeScript™ II 1st Strand cDNA Synthesis Kit (Vazyme, Nanjing, China). After screening for DEGs, four genes were selected for qRT-PCR based on their FPKM values [79]. The qRT-PCR assays were performed using the 7500 fast real-time PCR System (Applied Biosystems, Foster City, CA, USA) and Hieff^®^ qPCR SYBR Green Master Mix (Low Rox Plus) (Yeasen, Shanghai, China). *GhHIS3* was used as an internal control. The qRT-PCR conditions were as follows: an initial denaturation step at 95 °C for 5 min, followed by 40 cycles of denaturation at 95 °C for 10 s, annealing at 60 °C for 20 s, and extension at 72 °C for 34 s. The relative expression levels were calculated using the 2^−ΔΔCT^ method. The primers used in this experiment were designed using Primer Premier 5.0 (Table S6).

### 4.4. Detection and Analysis of Metabolites

The shoot apexes of CCRI50 and Guoxinmian11 at different developmental stages (a total of 18 samples, divided into 6 groups) were collected and placed on dry ice. The samples were then sent to Wuhan Metware Biotechnology Co., Ltd. (www.metware.cn, accessed on 2 September 2022) for the detection of widely targeted metabolites. Ultra-Performance Liquid Chromatography (UPLC, Shimadzu Corporation, Kyoto, Japan) and Tandem Mass Spectrometry (MS/MS, Applied Biosystems, Foster City, CA, USA) were used as the data acquisition systems.

CCRI50 was set as the experimental group, and Guoxinmian11 was designated as the control group. Based on the experimental design, sample collection and processing, metabolite extraction, and subsequent metabolite detection and analysis, metabolome data were obtained for metabolite identification and the quality control analysis of sample data, and DMs were screened to predict and analyze the functions of metabolites in the sample [80].

### 4.5. KEGG Enrichment Analysis

Identified metabolites were annotated using the KEGG Compound database (http://www.kegg.jp/kegg/compound/, accessed on 16 November 2022), and annotated metabolites were then mapped to the KEGG Pathway database (http://www.kegg.jp/kegg/pathway.html, accessed on 16 November 2022). The significant pathways were mapped to the Metabolite Sets Enrichment Analysis (MSEA), and their significance was determined by hypergeometric test’s *p*-values [81].

### 4.6. Orthogonal Partial Least Squares Discriminant Analysis

The Orthogonal Partial Least Squares Discriminant Analysis (OPLS-DA) combined Orthogonal Signal Correction (OSC) and Partial Least Squares Discriminant Analysis (PLS-DA) methods to identify differential variables by removing unrelated variations. After performing Log2 transformation on the raw data, centering was applied to standardize the data. In this analysis, X represented the quantitative information matrix of the samples, while Y represented the grouping information matrix of the samples. During the OPLS-DA modeling process, the X matrix was decomposed into two categories of information: one that was related to the grouping variable Y (the predictive components) and one that was unrelated to Y (the orthogonal components). The predictive components captured the variation in the data that correlates with the sample groups, while the orthogonal components represented noise or unrelated variations [82].

### 4.7. Principal Component Analysis, Hierarchical Cluster Analysis, and Pearson Correlation Coefficients

An unsupervised Principal Component Analysis (PCA) was performed by statistics function prcomp within R (www.r-project.org, accessed on 9 November 2022). The data were unit variance scaled before the unsupervised PCA [83].

The Hierarchical Cluster Analysis (HCA) results of samples and metabolites were presented as heatmaps with dendrograms, while Pearson correlation coefficients (PCCs) between samples were calculated by the cor function in R and presented as heatmaps. Both the HCA and PCC analysis were carried out by the R package Complex Heatmap. For the HCA, normalized signal intensities of metabolites (unit variance scaling) were visualized as a color spectrum.

### 4.8. DMs Selected

For the two-group analysis, DMs were determined by Variable Importance in Projection (VIP ≥ 1) and absolute Log2FC (|Log2FC| ≥ 1.0). The VIP values were extracted from the OPLS-DA result, which also contained score plots and per mutation plots, and was generated using R package MetaboAnalystR. The data were Log-transformed (Log2) and underwent centering before the OPLS-DA. In order to avoid overfitting, a permutation test (200 permutations) was performed.

### 4.9. Correlation Analysis Between Transcriptome and Metabolome

The association of transcriptomic data with metabolomic data in this multi-omics integrative analysis is detailed in Table S7, which outlines the correspondence between sample identifiers and groups named across different omics datasets. In this table, the “Sample” column represents the unified sample names, while the “Group” column denotes the unified group classification. The terms “Meta” and “Trans” refer to metabolomics and transcriptomics, respectively.

### 4.10. Vector Construction and Plasmid Transformation

To verify the function of the selected gene, an overexpression vector was constructed to obtain transgenic plants. The full-length open reading frame (ORF) of *GhTYDC-A01* was amplified and cloned into the plasmid pCAMBIA2300-HA under the control of the 35S promoter. The recombinant vector was then transformed into *Agrobacterium tumefaciens* strain GV3101 (Weidi, Shanghai, China) using the freeze–thaw method.

### 4.11. Arabidopsis Seedling Growth and Transformation

The *Arabidopsis* used in this study was the Columbia wild-type genetic background. *Arabidopsis* seeds were surface-sterilized by soaking in a 30% sodium hypochlorite solution for 10 min, followed by washing and air-drying. The seeds were then sown onto an agar-based medium. The culture plates were inverted and placed in the dark at 4 °C for vernalization for 2–3 days. After vernalization, the plates were transferred to a growth chamber at 22 °C with 60% relative humidity under a 16 h light and 8 h dark photoperiod. After 12 days of growth, the *Arabidopsis* seedlings were transferred to soil and grown until they reached full flowering. Flowering-stage plants were subjected to transformation using the floral dip method to generate transgenic *Arabidopsis* plants [84]. After infecting the *Arabidopsis*, the plants were passed on to subsequent generations, and phenotypic observations were made when they reached the T_3_ generation.

## 5. Conclusions

Flower bud differentiation in cotton is influenced and regulated by a variety of factors, and studying only a few specific genes or metabolites may not allow for a comprehensive understanding. In this study, early-maturing cultivar CCRI50 and late-maturing cultivar Guoxinmian11 were used as experimental materials. Transcriptomic and metabolomic analyses were conducted, and through the enrichment of DEGs and metabolites, we identified potential genes involved in the regulation of flower bud differentiation. A total of 616, 782, and 842 DEGs were detected between the two cultivars at three stages, respectively. And most of the DEGs were enriched in the biosynthesis of secondary metabolites and phenylpropanoid biosynthesis. The metabolomics analysis showed that metabolic pathways, biosynthesis of secondary metabolites, and phenylalanine metabolism played important roles in cotton flower bud differentiation. And phenolic acids might play important roles at the three development stages. The transcriptional and metabolic correlation analysis suggested that phenylpropane biosynthesis, tyrosine metabolism, and phenylalanine metabolism may be important metabolic pathways for cotton bud differentiation. And the gene *GhTYDC-A01* was identified in both the tyrosine metabolism and phenylalanine metabolism pathways. Overexpression of *GhTYDC-A01* in *Arabidopsis* showed a later flowering time compared to the wild type. This study lays the foundation for providing insight into the developmental mechanisms of flower bud differentiation in cotton and the future breeding of early-maturing cotton cultivars.

## Data Availability

The data supporting the findings of this study are available within the article and its Supplementary Materials. Additional data can be obtained from the corresponding author upon reasonable request.

## Supplementary Materials

The following supporting information can be downloaded at https://www.mdpi.com/article/10.3390/ijms26052277/s1.
