# Supplementary figures and images for: Exploring Metabolic Pathways and Gene Mining During Cotton Flower Bud Differentiation Stages Based on Transcriptomics and Metabolomics
Source: Int J Mol Sci. 2025 Mar 4;26(5):2277. doi: 10.3390/ijms26052277 (PMC11901054; doi:10.3390/ijms26052277)

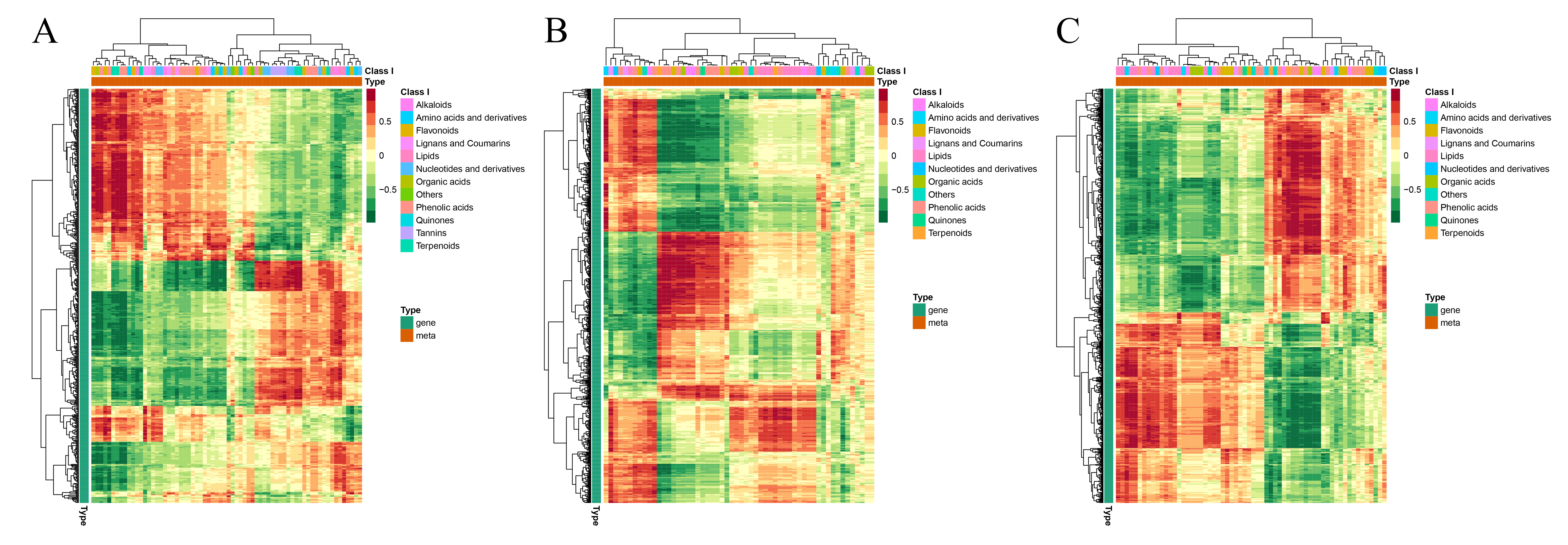

Supplement: Supplementary file 1 [file ijms-26-02277-s001.zip › Figure S1.png]
